# Large-scale detection of marine debris in coastal areas with Sentinel-2

**DOI:** 10.1016/j.isci.2023.108402

**Published:** 2023-11-08

**Authors:** Marc Rußwurm, Sushen Jilla Venkatesa, Devis Tuia

**Affiliations:** 1 Wageningen University, Geo-information Science and Remote Sensing Laboratory, Droevendaalsesteeg 3, Wageningen Gelderland 6708 PB, the Netherlands; 2 École Polytechnique Fédérale de Lausanne (EPFL), Environmental Computational Science and Earth Observation (ECEO) Laboratory, Route des Ronquos 86, Sion, Valais 1950, Switzerland

**Keywords:** Earth sciences, pollution, remote sensing

## Abstract

Detecting and quantifying marine pollution and macroplastics is an increasingly pressing ecological issue that directly impacts ecology and human health. Here, remote sensing can provide reliable estimates of plastic pollution by regularly monitoring and detecting marine debris in coastal areas. In this work, we present a detector for marine debris built on a deep segmentation model that outputs a probability for marine debris at the pixel level. We train this detector with a combination of annotated datasets of marine debris and evaluate it on specifically selected test sites where it is highly probable that plastic pollution is present in the detected marine debris. We integrate data-centric artificial intelligence principles by devising a training strategy with extensive sampling of negative examples and an automated label refinement of coarse hand labels. This yields a deep learning model that achieves higher accuracies on benchmark comparisons than existing detection models trained on previous datasets.

## Highlights

• A deep marine debris detector for Sentinel-2 images following data-centric AI principles
• Trained on a quantity-focused training set and quality-focused evaluation data
• Achieves state-of-the-art accuracy thanks to semi-automated data curation strategies
• Combinable with daily PlanetScope images to augment weekly Sentinel-2 observations

## Graphical abstract

**Figure:**
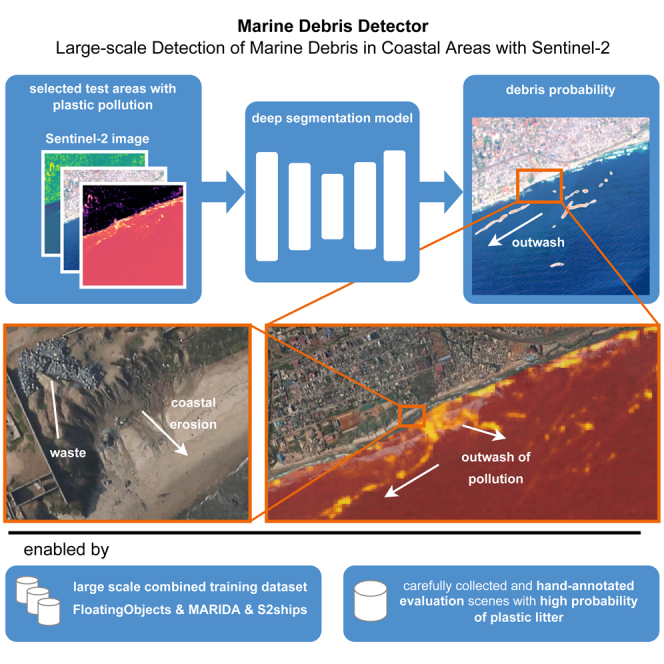

## Introduction

Marine litter is accumulating at alarming rates, with 19–23 million metric tons dispersed in 2016 alone.^1^ Plastic artifacts constitute 75% of marine litter, exceeding 5 trillion objects in numbers,^2^ and are causing a serious threat to marine ecosystems and human health. Approximately 80% of marine litter originates from terrestrial sources.^3^ It accumulates in rivers^4^^,^^5^ and lakes^6^ and eventually enters open oceans. Primary microplastics are purposefully manufactured to carry out a specific function, like abrasive particles or powders for injection molding. Secondary microplastics result from fragmentation of larger objects.^7^ In particular, transport in rivers causes macroplastics (>2.5cm diameter) to decompose into meso- (5mm−2.5cm) and microplastics (<5mm diameter),^7^^,^^8^ which then enter the food chain. Microplastics have been found across the entire planet and have been detected in antarctic penguins,^9^ deep-sea sediments,^10^ and human stool^11^ and have been shown to affect the growth of corals.^12^ A range of economic costs can also be associated with marine pollution, from clean-up expenses to loss of tourism revenue.^13^ It is clear that monitoring and mitigating water pollution is a major environmental, social, and economic challenge, and systematic mapping is needed to both identify pollutants and measure the success of awareness and clean-up programs. Continuous monitoring and litter quantification are often limited to individual surveys that are labor intensive and expensive to conduct regularly.^14^ These approaches can only cover a comparatively small area, even when surveyors are supported by aerial UAV imagery, as explored by several studies.^15^^,^^16^^,^^17^^,^^18^ Effectively, only a few developed countries, such as the United Kingdom, can afford a systematic monitoring program.^19^ These programs still require support from the local population in citizen science projects to collect ground data.^20^ This level of engagement requires a public sensitivity to the problem, awareness, and, eventually, the technological means to report pollutants.

Satellite imagery that provides data at reasonable spatial and high temporal resolution can support this monitoring in large marine areas.^8^ Even though it is a pressing issue, remote sensing-enabled monitoring of marine debris has only relatively recently emerged as a major research topic, as summarized by the broad reviews of Salgado et al.^21^ and Topouzelis et al.^22^ Both reviews compared drone, aircraft, and optical and radar satellite-based acquisition methods. In particular, machine learning models have been increasingly used for this problem, as summarized by Politikos et al.,^23^ who aggregated a comprehensive list of approaches and locations where machine learning algorithms have been deployed in the last years across the globe. For optical sensors, high spatial (<3m) and spectral resolutions beyond RGB (400nm to 2500nm) were found optimal for the detection of aggregations of marine debris. Synthetic aperture radar can be potentially suitable for detecting sea slicks^24^ that are associated with surfactants and change the surface tension of the water, which in turn reduces the radar backscatter. These slicks consist of microbial bio-films that can be connected with microplastics suspended in the sea-surface microlayer.^21^ However, a recent study^25^ demonstrated that only very high concentrations of microplastics lead to a sufficiently strong dampening of waves to be detectable with radar satellites. Similarly to sea slicks, macroplastics can aggregate in lines driven by environmental forces, such as wind speed, waves, or coastal fronts. For instance, windrows are accumulations of surface debris. Their geometry allows for efficient ship-based collection efforts, which can be highly effective, as demonstrated by Ruiz et al.^26^ Their collection campaign lasted 68 working days during the spring and summer of 2018 and gathered 16.2 tons of floating marine litter in the Bay of Biscay. This work demonstrated that detecting and collecting aggregated debris on the sea surface in geographic areas with a high pollution level can be directly attributed to macroplastic litter. Marine debris aggregations in windrows are sufficiently large to be detectable at medium resolutions of 10m by 10m achievable by Sentinel-2 and can effectively serve as a proxy for macroplastic litter in the oceans.^27^^,^^28^ However, further distinguishing floating objects of natural origins, such as driftwood or patches of algae and sargassum, from objects of human origins in large-scale medium-resolution imagery remains challenging and is an ongoing topic of current research.^29^^,^^30^^,^^31^^,^^32^ This further fine-grained distinction may require currently unavailable sensor technology^21^ and is beyond the scope of this work. Instead, we study the effectiveness of detecting heterogeneous marine objects of both natural and anthropogenic origins at a large scale with globally available Sentinel-2 imagery. In this work, we aim to monitor floating marine litter by detecting marine debris as a proxy at a large scale. To do so, we evaluate our detector in selected areas where it is likely that marine litter is present in marine debris due to local studies and reports in the news and social media. This evaluation strategy ensures that our detector is sensitive to plastic pollution if marine debris is detected. This work follows the principles of data-centric artificial intelligence,^33^ where the methodological innovation is concentrated on careful designing of the dataset rather than the specificities of the particular deep learning model.

Throughout this work, we will use the term *marine litter* according to the UN Environment Programme^34^ definition as *any persistent*, *manufactured*, *or processed solid material discarded*, *disposed of*, *or abandoned in the marine and coastal environment*. We use *marine debris* more broadly as *any aggregation of floating materials on the sea surface* that may or may not contain *marine litter* of anthropogenic origins. The terms “litter”, “debris,” and “plastic” have particular meanings to different groups of people depending on the scientific or technical context or cultural preference^7^ and “marine debris” is often, especially in US-English, used synonymously with “marine litter”. However, we believe a distinction is necessary for technical reasons in this application: visual inspection of the current satellite imagery (without on-site knowledge) cannot reliably distinguish marine litter of human origins from marine debris that may also be of natural origins. Hence, any work relying on hand annotations of satellite images cannot resolve this conflict objectively, as on-site knowledge of the composition of the visible marine debris is only available from dedicated campaigns^18^^,^^35^ that yield few thoroughly analyzed pixels. In prior work,^36^ we used the generic term “floating object”, while others like Booth et al.^37^ chose the term “suspected plastics”. Both terms entail their limitations by being either too broad, as “floating objects” may include ships, or are too focused on plastics over other forms of litter. Our definitions of anthropogenic *marine litter* and generic *marine debris* follow the practices of Kikaki et al.^38^ who annotated similar objects termed marine debris in the Marine Debris Archive (MARIDA) and are used consistently throughout this work.

The rest of the paper is organized as follows: The next section summarizes related work on detecting marine pollution with remote sensing technology. Section materials and methods describes training, validation, and evaluation data used in this study and details the implementation of the segmentation models in the marine debris detector. Section results presents results compared to related work and methodologies qualitatively and quantitatively. Further experiments test the robustness of the Marine Debris Detector concerning atmospheric correction and test the transferability to higher resolution PlanetScope imagery that can supplement the Sentinel-2 imagery used primarily in this work. The final Section discussion discusses the results and provides conclusions for future work.

## Related work

Detecting marine debris with satellite imagery at high (typically 3m to 7m with PlanetScope imagery) and medium resolution (mainly at 10m with Sentinel-2) is a rising scientific question in remote sensing research. Initial advances were made by pixel-wise classifiers using multi-spectral reflectance in combination with dedicated spectral indices, such as the Normalized Difference Vegetation Index (NDVI). Themistocleous et al.^39^ investigated the detection of floating plastic litter from space using Sentinel-2 imagery in Cypris and proposed plastic index as the ratio of near-infrared reflectance to the sum of red and near-infrared similar to NDVI. Similarly, Biermann et al.^40^ proposed a Floating Debris Index (FDI), which is a modification of the Floating Algae Index.^41^ They demonstrated the effectiveness of FDI with a naive Bayes classifier in two-dimensional NDVI-FDI feature space. However, this classifier, originally fitted on hand-selected training and evaluation data under optimal conditions, was not accurate enough on unfiltered satellite imagery in practice, as demonstrated by Mifdal et al.^36^ Kikaki et al.^38^ achieved the best accuracies with a pixel-wise random forest classifier that utilized the Sentinel-2 reflectance bands, a range of spectral indices, and textural features. Mifdal et al.^36^ investigated the suitability of learned spatial features with a convolutional neural network for binary marine debris detection. While their results showed general applicability toward detecting marine debris with deep segmentation models, they identified several limitations and the sensitivity to a range of false-positive detections that made their model not employable in an automated way. Simultaneously, Shah et al.^42^ annotated RGB PlanetScope imagery with bounding boxes and trained a deep object detector on the localization of marine debris. Most recently, Gomez et al.^43^ focused on detecting debris in rivers with Sentinel-2 and tested several deep segmentation models to understand and predict floating debris accumulations. Similar to this work, Booth et al.^37^ presents a supervised U-Net classifier named MAP-Mapper, which is learned on the MARIDA dataset aimed to predict the density of marine debris.

Several public datasets were made available alongside the respective publications. Both the FloatingObjects dataset^36^ and the MARIDA^38^ contain Sentinel-2 imagery with a substantial number of hand-annotations of visually detected marine debris hand-annotated. They differ mostly in the binary (debris vs. other, i.e., non-debris) and multiclass (types of debris) nature of the annotations. The NASA Marine Debris dataset^42^ focused on 3-channel RGB PlanetScope imagery with coarse bounding box annotations.

In this paper, we extend initial work of Mifdal et al.^36^ and train a deep segmentation model on the combined datasets of FloatingObjects^36^ and MARIDA.^38^ We further use additional datasets to train our detector, which we detail in the next section.

## Materials and methods

**Figure 1:**
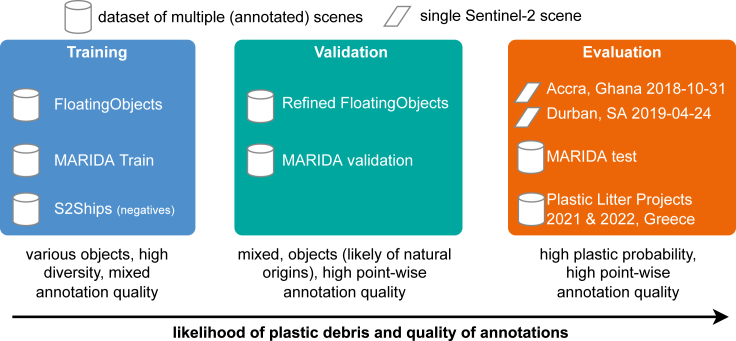
Schematic and motivation behind used datasets: we focus on quantity and diversity in the training datasets while prioritizing accurate annotations in validation and evaluation data The scenes in Accra and Durban likely contain plastic litter in the visible marine debris and are explicitly used for evaluation.

Defining and aggregating training data for marine debris detection is challenging due to the heterogeneous nature of objects, the novelty of the discipline, and the scarcity of available datasets. This section first outlines the sources, aggregation choices, and design decisions to generate the training, validation, and evaluation datasets used in this work. Specifically, Section training data focuses on the datasets used for training, while Section validation and evaluation sites outlines the validation and evaluation sets. An overview of the datasets is provided in Figure 1 alongside a table showing the number of marine debris and non-marine debris patches for each dataset in Table 1. For training datasets, we focused on quantity and aggregated a large dataset of heterogeneous marine debris and other floating materials alongside negative examples focused on ships (S2Ships). The quality of this large training pool is variable, but this also reflects the inherent difficulty of the task. In the validation and evaluation data, we focus more on the quality and accuracy of annotations of marine debris. The evaluation scenes were chosen explicitly in areas where we were certain, due to manual verification, that plastic pollution is present among marine debris. After describing the dataset, the models used are detailed in sections marine debris detector implementation and comparison methods, which describe our detector and the comparison methods, respectively. Accuracy metrics are described in Section evaluation metrics.

**Table 1:** Number of rasterized 128 px by 128 px image patches containing marine debris/non-marine debris

	MARIDA	FloatingObjects	Refined FloatingObjects	S2Ships
Training	930/1,087	19,587/19,607	–	0/29,833
Validation	616/569	–	868/1,583	–
Testing	270/602	–	903/1,294	–

Note that FloatingObjects contains overlapping image patches, which inflates the number compared to the non-overlapping MARIDA dataset.

### Training data

The available annotated data on the detection of marine debris are scarce. To our knowledge, only two publicly available datasets focusing on Sentinel-2 imagery are available today. The MARIDA^38^ provides multiple labels on polygon-wise hand-annotated Sentinel-2 images, and the FloatingObjects^36^ provides binary labels (floating objects versus water annotations) in coarse hand-drawn lines on Sentinel-2 scenes. We further improve the quality of these annotations by an automated label refinement heuristic defined for this problem. Our goal is to train a model that can predict marine debris from openly accessible satellite imagery in different conditions and, therefore make it possible to process both top-of-atmosphere and atmospherically corrected bottom-of-atmosphere data. For atmospheric correction, we further chose to use products corrected with Sen2COR^44^ that are readily available to download in Google Earth Engine rather than products corrected with ACOLITE,^45^ where the atmospheric correction would have to be done individually at each raw image scene. To study the effect of atmospheric correction, we test our models on imagery at different atmospheric processing levels (see Section role of atmospheric correction). To avoid confusion of marine debris with ships, one of the major problems highlighted in Mifdal et al.,^36^ we also include the S2Ships dataset^46^ that provides negative non-debris examples of class other. All three datasets are detailed in the next subsections.

#### Floating Objects

The FloatingObjects dataset originates from our prior work in Mifdal et al.^36^ and contains 26 different globally distributed Sentinel-2 scenes. Overall, 3297 floating objects were annotated by lines when visually identified as marine debris. In this work, we use this dataset exclusively for training, as a certain level of label noise is present in the annotations. Based on visual inspection and local expertise in Accra, we decided to exclude four regions accra 20181031, lagos 20190101, neworleans 20200202, and venice 20180630 to be re-annotated in the RefinedFloatingObjects validation dataset described later in Section RefinedFloatingObjects. The remaining 22 regions were used for training.

We follow the data sampling strategy of Mifdal et al.^36^ and crop a small image patch of 128px by 128px centered on each line segment of the available marine debris annotations. The 128px by 128px binary annotation map is rasterized from the polyline, and all pixels that touch the marine debris line are assigned a probability value of one, while all other background pixels are assigned a value of zero. To obtain negative examples without any marine debris, we select random points within the Sentinel-2 scenes and extract equally sized image patches. We also use both processing levels L1C (top-of-atmosphere) and L2A (bottom-of-atmosphere), where we always select the L2A image available in the Google Earth Engine Archive^47^ and resort to L1C if no atmospherically corrected image is available. The effect of atmospheric correction on the performance of the detector is evaluated later in Section role of atmospheric correction. In all cases, 12 Sentinel-2 bands are used. These are all the available bands, excluding the haze-band B10, which the Sen2COR atmospheric correction^44^ algorithm removes automatically.

##### Label refinement module

**Figure 2:**
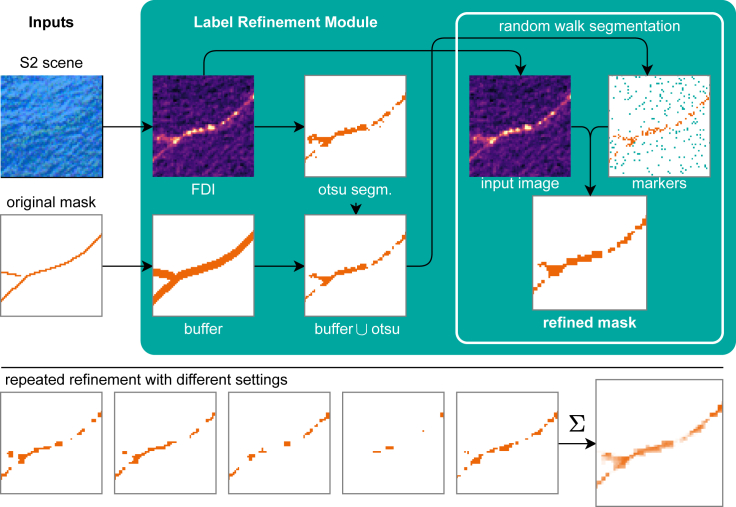
Label refinement module for the FloatingObjects dataset It inputs a Sentinel-2 image and the original hand annotation of the FloatingObjects dataset (left). An Otsu-threshold segmentation buffered around the hand labels^48^(center) is used to sample marker points (shown on the right) for a random walk segmentation algorithm^49^ that results in a refined annotated mask (right). By varying parameters, we generate 24 different variants of the mask, whose average expresses the uncertainty and fuzziness on the borders of the debris (second row).

While the FloatingObjects dataset provides many labels, the annotated lines are often too simple and do not capture the width and geometry of the underlying marine debris. We improve the hand annotations by an automated label refinement module that generates a mask that reflects more closely the geometry of the debris in the proximity of the line annotations (Figure 2). The module inputs a Sentinel-2 scene and the original line annotations mask. In the first stage (left side of Figure 2), we buffer the hand-annotated line to obtain a region of potential marine debris. Then, we calculate the FDI using the Sentinel-2 scene and perform a segmentation of the FDI image with an Otsu threshold.^48^ The buffer and segmentation are then combined to obtain a preliminary area of marine debris in the vicinity of the original annotations. In the second stage, we randomly sample potential marine debris pixels, as well as markers for non-debris pixels (class other) in the remaining parts of the image. These markers are the starting points of a random walk segmentation algorithm,^49^ which is a fast algorithm that requires a few labeled pixels as markers. The markers are assumed to be accurately annotated, while the pixels between the markers are uncertain and are then annotated by an underlying anisotropic diffusion process that ensures that homogeneous areas are assigned to the same class. Crucially, one set of parameters (homogeneity criterion, buffer size, and marker sampling frequency) of the random walker algorithm leads to one potential debris map. Therefore, we vary those parameters and average all maps to capture the underlying undefinedness of the borders of marine debris, as shown in the bottom row of Figure 2. We choose randomly among two homogeneity criteria, three buffer sizes, and four marker sampling frequencies, resulting in 24 possible variations. Considering the original mask among the candidates, the model is trained to reproduce one of these 25 potential label maps. Across multiple epochs, this captures the edge-undefinedness of the debris patch.

#### MARIDA

The MARIDA was collected by Kikaki et al.^38^ for developing and evaluating machine learning algorithms for marine debris detection. MARIDA contains 63 temporally overlapping Sentinel-2 scenes from 12 distinct regions. In total, 6,672 polygons were annotated, of which 1,882 are marine debris and 2,447 marine water. The remaining 2,343 polygons are annotated in one of 13 further classes with between 24 and 356 annotations each that we do not use in this study.

We use MARIDA as an additional training, validation, and evaluation data source, but consider only patches annotated as marine debris (positive class) and treat instances of marine water as negatives. In other words, the dataset is reduced to a two-classes problem. The original MARIDA dataset contains Sentinel-2 imagery with 11 bands that have been atmospherically corrected with the ACOLITE^50^ algorithm. In this work, we use re-downloaded and Sen2COR atmospherically corrected MARIDA scenes with 12 bands, as we want to apply our detector on 12-band Sentinel-2 imagery that had been atmospherically corrected with Sen2COR,^44^ as is readily available, for instance, in Google Earth Engine.^47^ This avoids reprocessing additional imagery after download and simplifies the application on new scenes. To harmonize this dataset, we re-downloaded all Sentinel-2 scenes from Google Earth Engine to retrieve 12-band imagery for MARIDA compatible with the other datasets. Like FloatingObjects, we use the atmospherically corrected L2A Sentinel-2 imagery whenever available. We also excluded one scene near Durban from MARIDA (named S2 24-4-19 36JUN) to avoid spatial overlap, and potential positive biases with our evaluation scene described later in Section validation and evaluation sites.

#### Sentinel-2 Ships

Ships and their wakes can cause false-positive predictions of marine debris, as reported by Mifdal et al.^36^ We decided to explicitly add images of ships without any annotated marine debris as negative examples. We use the Sentinel-2 Ships (S2Ships) dataset of Ciocarlan et al.,^46^ which segmented ships with Sentinel-2 imagery. In our training pipeline, we retrieve these ship positions, load an image centered on each ship, and show it to our detector during training with a negative prediction mask indicating the class other.

### Validation and evaluation sites

For finding the best neural network design and hyperparameters (i.e., validation), as well as for the final independent evaluation, we used datasets with high-quality annotations. For both sets, we combine the MARIDA datasets, according to their validation and evaluation partitioning schemes, with a refined version of the FloatingObjects dataset that we describe in the next Section refined floating objects. For further qualitative evaluation, we additionally use imagery from the Plastic Litter Projects 2021 and 2022, detailed further in Section plastic litter projects. For both validation and evaluation datasets, we focus on using accurate annotations and we select only sites with a high probability of plastic pollution specifically for final evaluation, as detailed further in the next sections.

#### Refined Floating Objects

We create a refined version of the FloatingObjects dataset (Section floating objects) with less label noise by re-annotating some a subset of FloatingObjects regions by individual point locations of which we are certain that they are localized accurately on visible marine debris in the imagery. We conduct this annotation in Google Earth Engine^47^ and select the subset of regions named lagos 20190101, neworleans 20200202, venice 20180630, and accra 20181031. We also included two new areas, which are marmara 20210519 and durban 20190424. By carefully annotating these areas, we are confident that we captured the precise location of the class marine debris in these Sentinel-2 scenes. To train a model, we also need examples for the negative other class to calculate accuracy scores that capture a diverse set of negatives, like open water, land, coastline, and ships, that likely confuse the model. To obtain these negative examples, we iteratively added negative examples by monitoring the result of a smileCART^51^ classifier implemented online in Google Earth Engine. This classifier serves as a proxy antagonist to us as labelers, i.e., it will highlight areas that appear like marine debris and will be checked by annotators. We explicitly added new negative examples in locations where this proxy classifier incorrectly predicted marine debris. Hence, we captured meaningful negative point locations of the other class that was difficult to distinguish from the annotated marine debris by the smileCart classifier.

At validation and evaluation time, we extract a 128px×128px patches centered on each of these annotated points that are labeled as either marine debris (positive) or other (negative). We can only be certain about the class at the precise annotations of the point in the center of each image patch. Hence, we first segment the entire patch using the semantic segmentation model but then extract the prediction only at the center pixel corresponding to the annotated point for accuracy estimation. This selection effectively simplifies the segmentation problem to a classification problem at the center of the image patch. It allows us to use standard classification metrics to measure the accuracy (described in Section evaluation metrics).

Among the six regions in this dataset, we use the Sentinel-2 scenes lagos 20190101, neworleans 20200202, venice 20180630, and marmara 20210519 for validation, as we are not certain about the composition of the visible marine debris in these images. For instance, marmara 20210519 likely contains floating algae (sea snot), as it coincides with reported algae blooms^52^ which are often present in this area.^53^ We use the accurate annotations of this generic marine debris in these areas to calibrate the model hyperparameters, such as the classification threshold, before final evaluation.

For evaluation, we use the scenes accra 20181031 and durban 2019042, as these areas very likely contain plastics in the marine debris.

**Figure 3:**
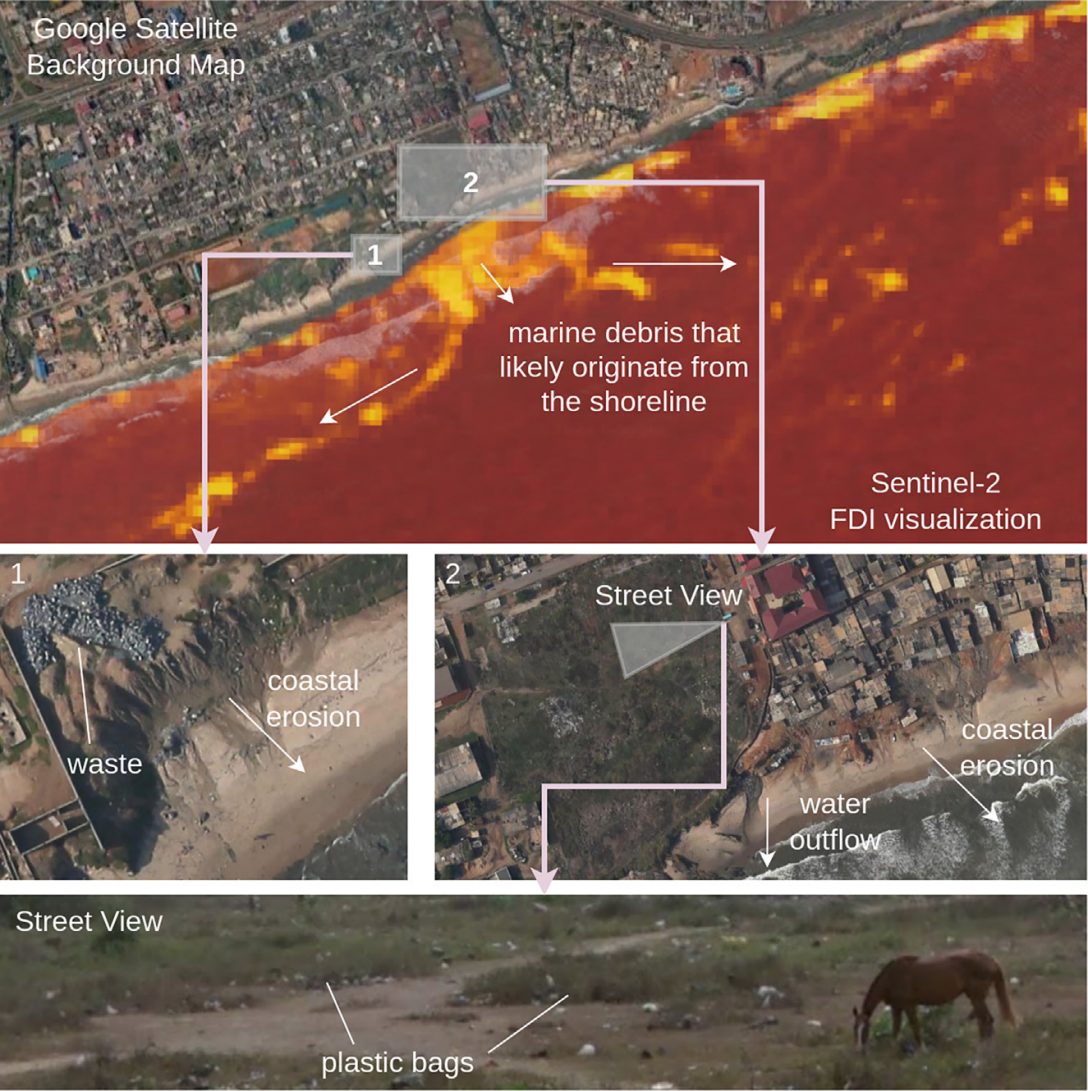
Evaluation scene in Accra, Ghana The top row shows an FDI visualization of the Sentinel-2 image of October 31^st^ 2018, where marine debris is washed into the open waters. Closer investigations with high-resolution satellite images (center row) show that coastal erosion is present, and this area is generally polluted with human litter. This is also confirmed by a Google Street View image shown on the bottom row.
(2) **Evaluation Scene Durban, South Africa, 2019-04-24.** This evaluation scene was first identified by Biermann et al.,^40^ who used social media and news reports to select areas of plastic pollution. It covers marine debris that likely contains plastic litter from a flood event in Durban following heavy rainfall starting on April 18^th^ 2019. This flood discharged large quantities of debris into the harbor of the Durban Metropole, as shown in Figure 4. We acquired one Sentinel-2 image from April 24th, shown in Figure 4C, where visible debris originates from the harbor area (highlighted in gray). The debris in this image likely contains plastic litter. This image is particularly difficult to predict, as clouds and haze from former precipitations are still visible in this scene. The patches of marine debris visible in the FDI representation are less pronounced than in the Accra scene, which has more clearly identifiable objects. In this area, both top-of-atmosphere (L1C) and bottom-of-atmosphere (L2A) Sentinel-2 images are available. We compare the model performance on both versions later in Section role of atmospheric correction.

(1) **Evaluation Scene Accra, Ghana, 2018-10-31.** Beach surveys in 2013 showed that plastic materials made up the majority of 63.72% of marine debris washed onto evaluated beaches.^14^ A recent study^54^ estimated the daily plastic mass transport of plastic in the Odaw river running through Accra into the sea between 140 and 380 kg per day. Qualitatively, one particular area in this Sentinel-2 scene, shown in Figure 3 (top), shows an outwash of debris from the coast. In this image, the marine debris is visible in yellow (high FDI). We show a high-resolution background map from Google Satellites for land and shoreline to provide a reference. Two zoomed-in areas (named 1 and 2 in Figure 3) show that coastal erosion is visible alongside waste and sewage outflows aggregations. Finally, a Google Street View image (bottom row of Figure 3) further confirms this area’s general pollution level. Only a Sentinel-2 image at the top-of-atmosphere processing level (L1C) is available in Google Earth Engine in Accra.

**Figure 4:**
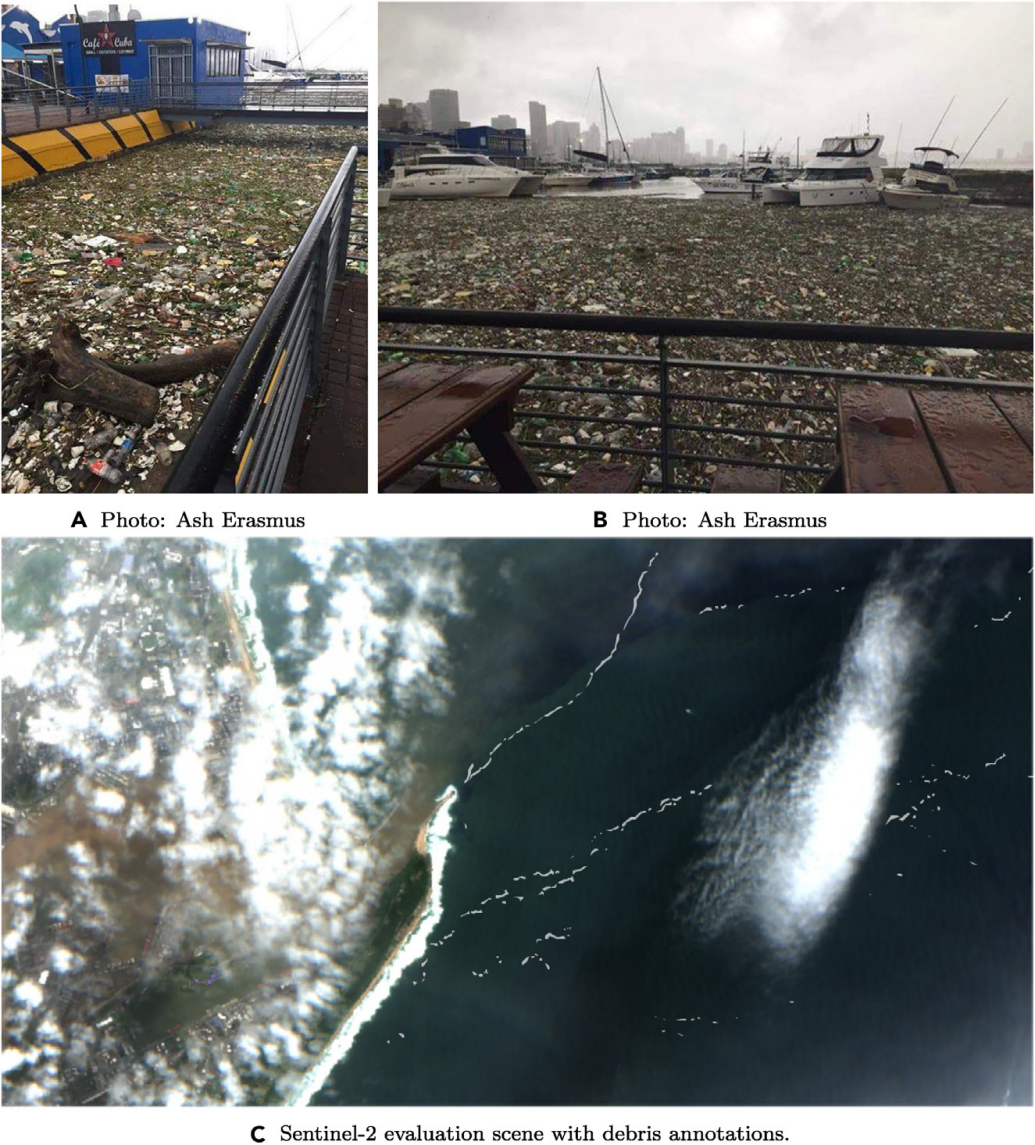
Evaluation scene from Durban, South Africa Additional imagery shared by local news and social media (top row) shows the level of plastic pollution on 24^th^ of April 2019. The Sentinel-2 image (bottom image) shows the corresponding Sentinel-2 scene we use for evaluation. Photos (A) and (B) are taken in Durban harbor and show large-scale pollution of plastic litter. (C) shows the Sentinel-2 images of the same day with patches of debris being washed into the Indian Ocean. These patches are annotated for visibility and are less visible in the image itself.

#### Plastic Litter Projects

The third evaluation area covers Sentinel-2 data showing explicitly deployed debris targets in the Plastic Litter Projects of 2021 and 2022^18^^,^^35^^,^^55^ on the island of Lesbos, Greece. In 2021, one 28m diameter high-density polyethylene (HDPE) mesh was deployed on June 8^th^ 2021, followed by a 28m wooden target on June 17^th^ 2021. Both were visible during 22 Sentinel-2 satellite overpasses until 7^th^ of October 2021. In the Plastic Litter Project 2022, one 5m × 5m inflatable PVC target, alongside two 7m diameter HDPE meshes were deployed on June 16^th^ 2022. One HDPE mesh was cleaned regularly, while the other was subject to natural fouling and algae. The objects were deployed until the 11^th^ of October 2022 and were visible in 23 Sentinel-2 acquisitions. Additional smaller $1m^{2}$ and $3m^{2}$ targets were also deployed throughout the project phase to study visibility and the material’s decomposition in water but were too small to be visible in the Sentinel-2 scenes. We use the Sentinel-2 data of the 2021 campaign to qualitatively test the ability of our detector and comparison models to detect the deployed targets in the Sentinel-2 imagery.

### Marine debris detector implementation

This section describes the implementation of the Marine Debris Detector as a deep segmentation model that inputs a 12-channel Sentinel-2 image and estimates the probability of marine debris’s presence for each pixel.

#### Segmentation model architectures

**Figure 5:**
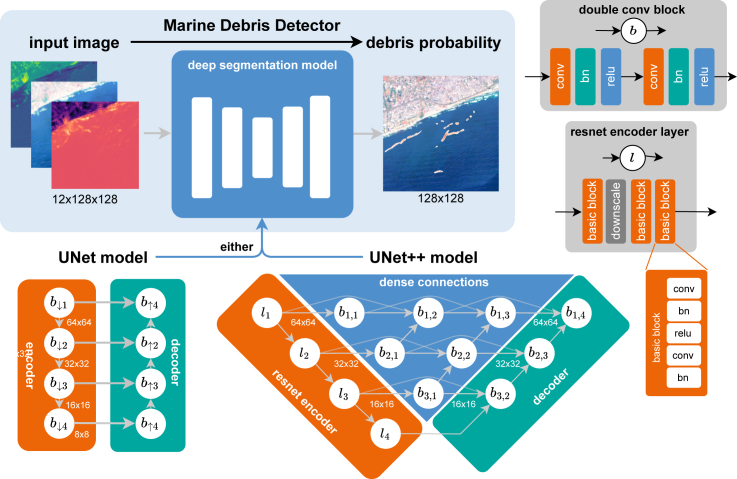
Schematic of the Marine Debris Detector implementation with an underlying Unet^56^ or Unet++^57^ segmentation model A 12-channel input image (top-left) is encoded to hidden feature representations in several levels of resolution (vertical pathways) and decoded to a probability of marine debris (top-right). Higher resolution pathways ensure that the resulting segmentation map is fine grained, while lower resolution encodes global information on the entire scene. Unet++^57^ extends the original Unet^56^ by adding additional dense connections in the skip pathways indicated in blue.

We implemented the UNet^56^ and Unet++^57^ architectures, as shown in Figure 5. The Unet segmentation model of Ronneberger et al.^56^ was developed for medical image segmentation and is heavily used in remote sensing due to the fine-grained segmentation masks it can produce. The success of the Unet is strongly related to its early skip connections, which help maintain the details of the image in the final map. As such, skip connections enable the propagation of a high-resolution representation of the input image through the entire network. This network was the one used previously by Mifdal et al.^36^ for marine debris detection.

**Figure 6:**
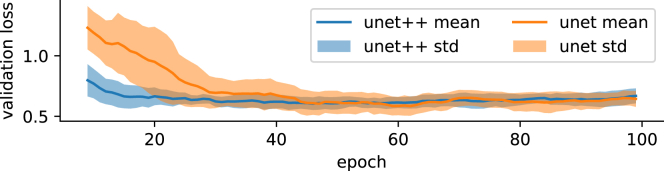
Validation loss during training of three Unet++ and Unet models The Unet++ finds an optimum earlier and has less variance (shown in 1σ standard deviation of 5 model runs) between the models in the early stages of training.

The Unet++^57^ variant extends the original Unet by replacing the original encoder with a ResNet^58^ with four blocks (indicated as $l_{i}$). ResNets are the *de facto* standard feature extractor in computer vision, as they can learn complex representation while requiring fewer weights than many earlier networks. The decoder consists of three double-convolutional blocks (indicated with $b_{i}$). Each block consists of two convolution-batchnorm-relu transformations. While the original Unet directly connects the output of each encoder layer with the corresponding decoder layer of same resolution, the Unet++ adds additional double-conv blocks in these skip pathways that are connected densely in the spirit of DenseNet neural networks.^59^

### Comparison methods

We compare models trained within our training framework to approaches from recent literature. In particular, the Unet trained by Mifdal et al.^36^ on the original FloatingObjects dataset, and a random forest classifier, denoted by rf, trained on the original MARIDA dataset.^38^ For the Unet, we use the provided pre-trained weights for their model. Similarly to our segmentation models, we also determine the best classification threshold based on the validation set to achieve results with balanced precision and recall, which is 0.039. For the rf, we train the random forest on 11 Sentinel-2 bands, as in the original paper with 12 output classes, and combine the predictions into a binary scheme by considering marine debris as the positive class and treat all other 11 non-debris classes as other. In the results section, we denote these two models as Unet and rf and indicate that they have been trained on the “original data” of their respective papers.

We also train the random forest on the combined training dataset described in Section training data, which we denote as “trained on our dataset”. For the random forest, we use an identical feature extraction pipeline as described in Kikaki et al.,^38^ which results in 26 features containing the original spectral bands, spectral indices, and textural features. As the random forest is a pixel-wise classifier, we treat each pixel separately and create a roughly balanced training pixel dataset set from our image training dataset. We select five positive pixels (annotated as marine debris) and five negative other pixels from each image. This results in 70 000 training pixels. As for the other comparison approaches, we tune the classification threshold based on the validation dataset, which is 0.663.

### Evaluation metrics

We compare all models trained on “original data” and “our dataset” on several metrics on the evaluation sets of Durban, Accra, and the MARIDA test partitions.

(1) We include the overall accuracy ratio of correct classifications to total samples. It is straightforward to interpret, but susceptible to class imbalance. Our selected validation and evaluation sets, however, have a general balance between positive and negative samples.
(2) f-score is the harmonic mean between precision and recall that, in contrast to individual precision and recall scores, is more robust to the choice of the classification threshold.
(3) The area under the receiver operator curve (auroc) is a metric that is independent of the classification thresholds but easily saturates for relatively accurate classifiers with values close to 1.
(4) The jaccard index, also known as intersection over union, is commonly used for object detection and measures the number of intersections of two sets (predictions and ground truth) divided by their union.
(5) The kappa statistic compares two classifiers: the model and a randomly guessing baseline. Values of zero indicate that the tested model is not better than a random baseline, while positive correlations indicate that the tested model outperforms the trivial baseline.

Higher values are better for all metrics, and values of 1 indicate a perfect score.

## Results

We first compare the models quantitatively and qualitatively in Section numerical comparisons. We then predict one entire Sentinel-2 scene (Durban) in Section role of atmospheric correction and quantify the false-positive predictions on both bottom-of-atmosphere and top-of-atmosphere Sentinel-2 imagery. In the final experiment Section transferability to PlanetScope resolution, we test how a re-trained 4-channel detector can predict marine debris on higher resolution PlanetScope imagery, which can complement Sentinel-2 imagery in practice.

### Numerical comparisons

Table 2 shows the quantitative results of rf and Unet models trained on the respective original data in comparison to rf, unet, and unet++ trained with our training setting on the combined training dataset and refinement strategies described in Section training data. We see that models trained in our combined training framework achieve the best accuracy metrics in all experiments including those where the label refinement is not used (column “no-ref”). As expected, the deep-learning-based UNet and the Unet++ models outperform the pixel-wise random forest classifier. This is likely due to the advantage of convolutional neural networks to learn spatial patterns within their convolutional perceptive field. Both Unet and Unet++ achieve equal accuracies within one standard deviation on the Marida test set, while the Unet++ achieves a better accuracy on the Durban and Accra scenes. The label refinement module also improves the Unet++ performance on Marida test and Durban. However, on Accra, the best scores are achieved with an Unet++ model without refinement module (indicated by “no-ref”). For the remaining paper, we use the Unet++ model in the Marine Debris Detector, as it has fewer parameters and finds an optimum earlier and more consistently between random seeds (1σ standard deviation shown) than the Unet in the training process, as shown in Figure 6.

**Table 2:** Quantitative comparison of models trained on original data (rf^38^, Unet^36^), versus models trained on the training data compiled in this work

Trained on	Original data		Our train set			
	rf	unet	rf	Unet	Unet++	Unet++ no-ref
**Accra**						

ACCURACY	0.653	0.882	0.680	0.924 ± 0.016	0.930 ± 0.016	**0.948**± 0.008
F-SCORE	0.464	0.871	0.545	0.920 ± 0.018	0.926 ± 0.018	**0.948**± 0.008
AUROC	0.246	0.965	0.899	0.978 ± 0.008	0.981 ± 0.006	**0.989**± 0.005
JACCARD	0.302	0.772	0.374	0.852 ± 0.030	0.862 ± 0.031	**0.900**± 0.014
KAPPA	0.301	0.764	0.357	0.848 ± 0.031	0.859 ± 0.031	**0.897**± 0.017

**Durban**						

ACCURACY	0.781	0.587	0.811	0.908 ± 0.010	**0.934**± 0.018	0.905 ± 0.011
F-SCORE	0.105	0.497	0.708	0.756 ± 0.032	**0.837**± 0.053	0.776 ± 0.026
AUROC	0.376	0.765	0.862	0.850 ± 0.030	**0.914**± 0.018	0.886 ± 0.053
JACCARD	0.055	0.330	0.548	0.609 ± 0.042	**0.722**± 0.048	0.635 ± 0.034
KAPPA	0.082	0.245	0.569	0.704 ± 0.037	**0.797**± 0.063	0.717 ± 0.031

**Marida test set**						

ACCURACY	0.697	0.838	0.811	**0.865**± 0.006	**0.867**± 0.005	0.851 ± 0.006
F-SCORE	0.288	0.701	0.708	**0.741**± 0.012	**0.749**± 0.009	0.710 ± 0.015
AUROC	0.488	0.764	0.862	**0.738**± 0.012	**0.746**± 0.021	0.733 ± 0.006
JACCARD	0.168	0.539	0.548	**0.589**± 0.015	**0.598**± 0.012	0.551 ± 0.018
KAPPA	0.197	0.593	0.569	**0.654**± 0.016	**0.661**± 0.012	0.615 ± 0.017

We also test an Unet++ model without label refinement module, indicated by the “no-ref” suffix in the last column.

**Figure 7:**
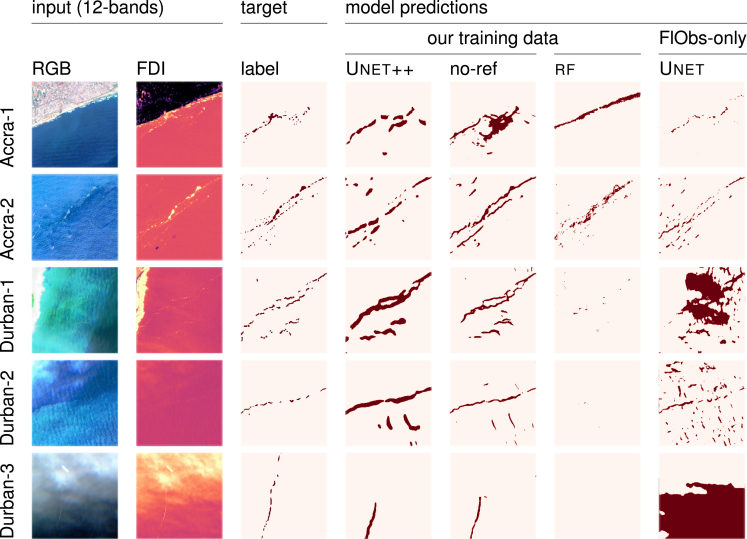
Qualitative predictions of the three models on images covering each 2.56 km by 2.56 km from the Accra and Durban sets Our Unet++ produces *marine debris* predictions similar to the hand annotations (target/label) with the fewest false positives. An interactive qualitative comparison is available under https://marcrusswurm.users.earthengine.app/view/marinedebrisexplorer.

Figure 7 compares models qualitatively on selected 256px × 256px each patches covering 2.56km by 2.56km. The tiles are from the Accra and Durban evaluation scenes, where it is highly plausible that plastic pollution is present in marine debris. We compare the Unet++ model with and without label refinement, the random forest rf with features of Kikaki et al.,^38^ trained on our dataset, and the Unet from Mifdal et al.^36^ trained on the original FloatingObjects (FlObs) dataset only. The first two columns show RGB and FDI representations of the multi-spectral Sentinel-2 scenes. The third column shows hand-annotated masks (shown in red). We generally see the quantitative results mirrored in these qualitative examples, where the deep learning model trained on our combined training set produces the most truthful masks of floating marine debris. While none of the models captured the hand annotations perfectly, the Unet++ produced the visually most accurate predictions with the fewest false positives across most evaluation scenes. The Unet++ without label refinement (indicated by “no-ref”) provides generally thinner predictions than the Unet++ with refinement module, which we connect to the refinement module always enlarging the target mask of marine debris to some degree during training. In Accra-1, Unet++ and Unet^36^ capture the general location of the objects, while the random forest rf^38^ detected natural waves along the entire coastline as marine debris. The Unet++ without refinement module appears to merge multiple patches of debris here and does not accurately capture the individual objects. Accra-2 shows several sargassum patches in between ships. Generally, all models predict these patches well, while still some ships are confused with marine debris. The Durban scenes are more challenging and show more atmospheric perturbations through clouds and haze. The Unet++ predicts the general locations of the annotated marine debris well until the cloud coverage is too dense, as seen in Durban-3. The original Unet^36^ predicts a large number of false positives, which was also stated as a limitation in their original work. The random forest rf of Kikaki et al.^38^ tends to under-predict the marine debris in all three Durban scenes and only identifies a few individual floating object patches in Durban-1.

**Figure 8:**
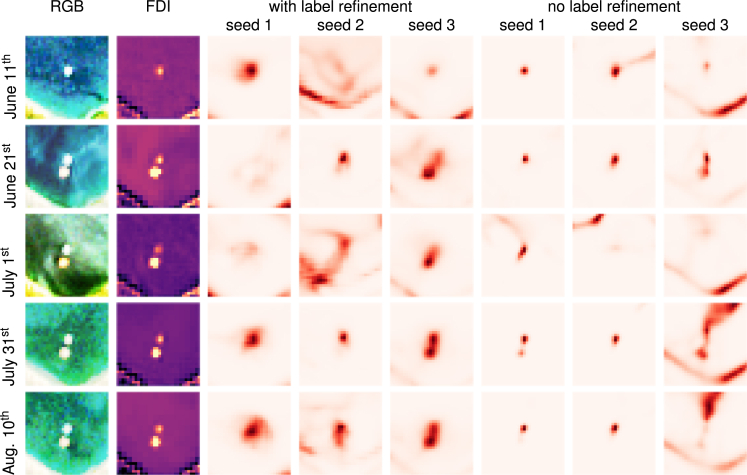
Classification probabilities for Sentinel-2 scenes of deployed targets in during the Plastic Litter Projects 2021 All models assign higher probabilities to the deployed targets. Still, only few models detect both targets. Other pixels, such as coastlines, are sometimes assigned a higher marine debris probability. Models trained with the label refinement module tend to predict larger patches with less spatial detail.

Finally, we compare different Unet++ models trained on different initialization seeds, with and without label refinement on images of the Plastic Litter Projects 2021^18^ (Figure 8). Most models capture the general location of the deployed targets on all scenes. However, some models (seed 3; no label refinement and seed 2 with label refinement) confuse the coastline and some water areas for marine debris. Seed 1 with label refinement appears to miss the deployed targets on June 21^st^ and July 1^st^, similarly to the model trained on seed 2 with label refinement on July 1^st^. Similarly to the previous result, models trained with refined labels predict larger but also less defined patches compared to models trained without. This experiment demonstrates the challenges associated with detecting individual objects that span only few pixels. However, we would like to highlight that these deployed targets are not representative of the marine debris seen in open waters, on which the models have been trained on. These objects typically form long lines rather than round shapes, and we believe that the difference in geometrical shape, rather than spectral appearance, is a major feature that the deep learning models use for their predictions.

### Role of atmospheric correction

In this experiment, we follow a realistic deployment scenario and predict the entire Durban scene of 3122px × 3843px with the Unet++ model in overlapping 480px × 480px patches. We then consider pixels predicted with a probability higher than the prediction threshold and treat each local maximum as a marine debris detection. We set a minimum distance of 3px between local maxima to avoid marine debris detections being too close to each other. Furthermore, we compare predictions of the same model using either a top-of-atmosphere (TOA) Sentinel-2 scene or a bottom-of-atmosphere (BOA) atmospherically corrected Sentinel-2 scene, to assess the effect of atmospheric correction on the model predictions.

**Figure 9:**
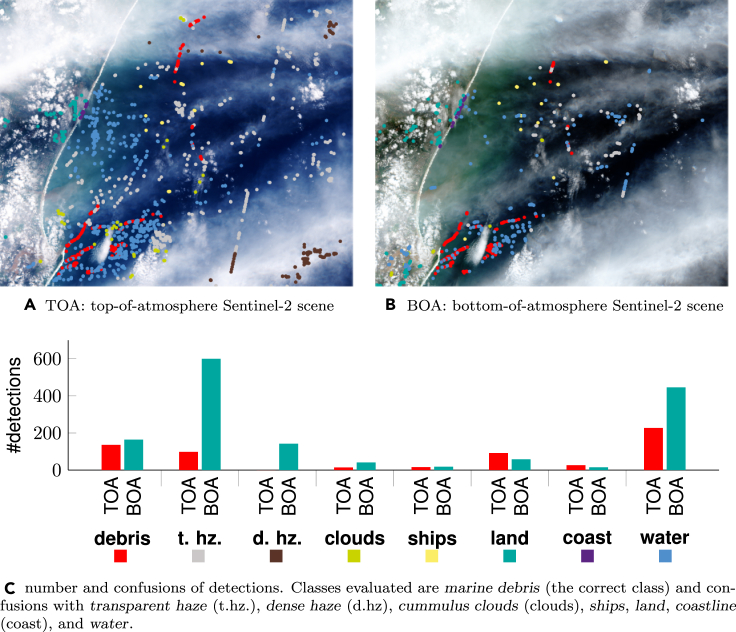
Analysis of confusions of detections in atmospherically corrected bottom-of-atmosphere (BOA) and not correction top-of-atmosphere (TOA) Sentinel-2 imagery of the Durban scene In (A) and (B), detections are colored according to the classes of (C).

We show both images alongside the locations of detections (scatter points) in Figures 9A and 9B, respectively. The red scatter points indicate correctly detected marine debris. Points of other colors indicate false positives with other classes’ transparent haze (t.hz.), dense haze (d.hz), cumulus clouds (clouds), ships, land, coastline (coast), and water, alongside marine debris (debris). Figure 9C further shows a quantitative summary of the confusion between classes. We generally see a comparable number of marine debris detected at both BOA (136 detections) and TOA (164 detections) processing levels. This shows that the classifier is sensitive to marine debris in both top-of-atmosphere and bottom-of-atmosphere satellite imagery. However, predictions based on top-of-atmosphere data had more false-positive predictions leading to a lower precision. This is especially visible in the t.hz. and d.hz categories as well, as in water, as shown in the bar plot of Figure 9C. Overall and not shown in the figure: 609 objects were detected in the BOA scene, and 1484 objects as marine debris in the top-of-atmosphere scene. For comparison, the Unet trained only on the FloatingObjects dataset of Mifdal et al.^36^ detected 20 830 objects in the BOA scene and 33 665 at TOA processing level, which is more than one order of magnitude more false-positive predictions compared to the Unet++ shown in Figure 9. This demonstrates even more the importance of compiling larger and more precise training datasets with a rich pool of negative examples that account for objects easily confused with marine debris. It demonstrates the current limitations and general difficulty of detecting marine debris automatically on Sentinel-2 imagery with the current technology. The extreme imbalance between a very low number of marine debris pixels (if any) and everything else visible in the Sentinel-2 scene poses a severe challenge to the automated detection of marine debris. Overall in this experiment, only 6448 of 11 997 846 pixels were annotated as marine debris, which represents coverage of only 0.05%. In this circumstance, identifying less than potential 1,000 objects in a 31km by 38km is an achievement and allows to validate these detections visibly with limited manual effort in practice. This work can be further reduced by additional targeted post-processing by masking clouds, land, and shoreline explicitly, which we consider outside of the scope of this work.

### Transferability to PlanetScope resolution

In this final experiment, we test how well the Unet++ model trained on Sentinel-2 imagery can predict on PlanetScope without being fine-tuned on PlanetScope imagery specifically. For this experiment, we had to downsample the PlanetScope imagery from 3m to 5m as the resolution gap between trained 10m resolution and full 3m PlanetScope imagery was too large. On the original resolution, the model created artifacts in the predictions, which disappeared at downsampled 5m PlanetScope imagery. For the Sentinel-2 image, we use the same model with 12 input channels as in the previous experiments. For the 4-channel PlanetScope imagery, we re-trained the Unet++ model on the identical Sentinel-2 training data but removed all spectral bands except B2, B3, B4, and B8 for RGB+NIR. This 4-channel model achieves a slightly worse validation accuracy (0.01–0.03 in f-score) than the 12-channel model. This slight decrease in accuracy also indicates that the four high-resolution 10m bands are the most informative for marine debris detection, which is reasonable given the small size of debris and previous literature.^40^

**Figure 10:**
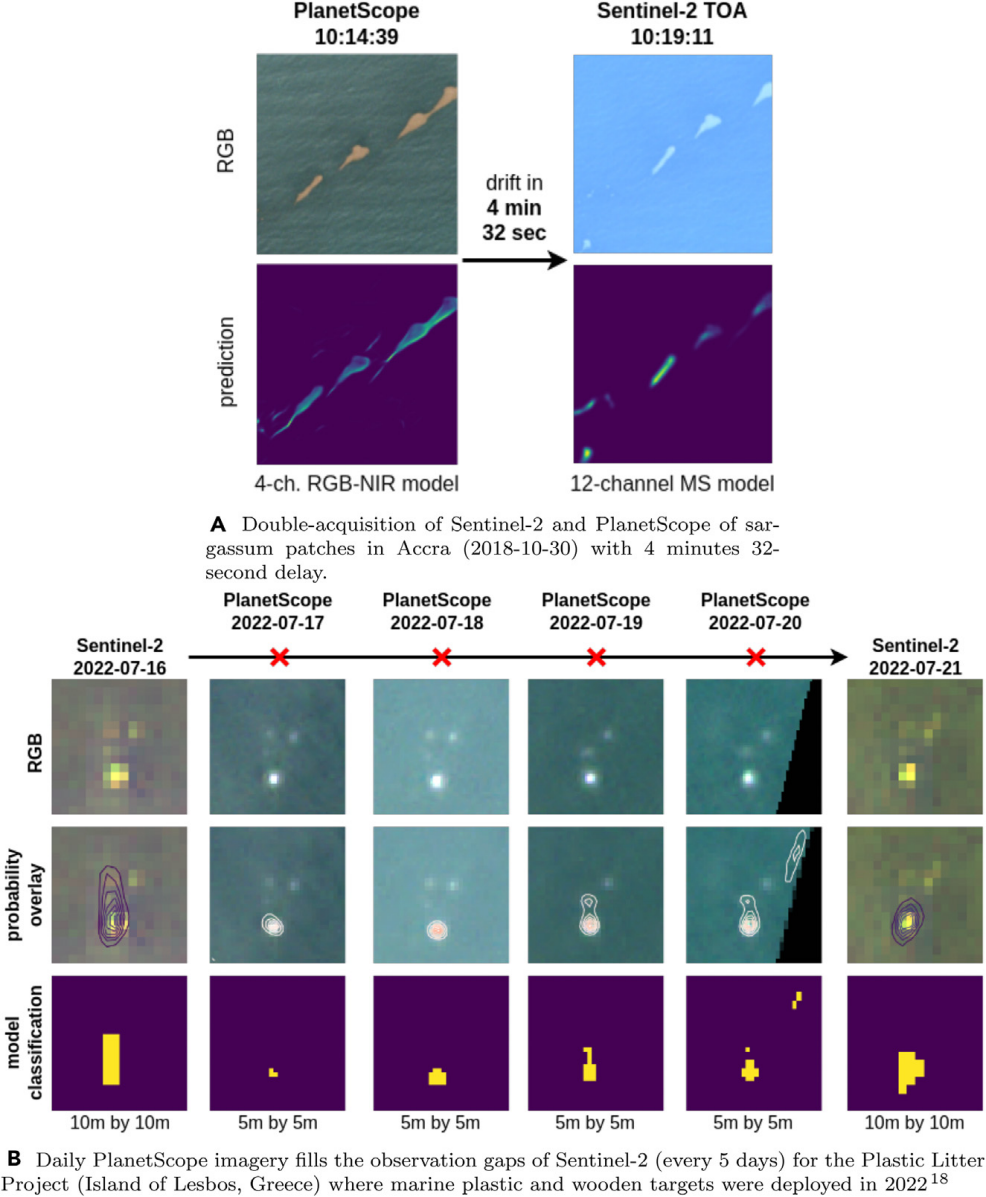
A four-channel RGB+NIR model trained on Sentinel-2 imagery can classify marine debris in 5 m × 5 m downsampled PlanetScope images, while being trained on 4-channel Sentinel-2 imagery We showcase two use cases. In (A), a simultaneous acquisition of S2 and PS in Accra shows the drift direction of Sargassum patches. In (B), PlanetScope images augment S2 observations in the Plastic Litter Project.

We consider two use cases in Figure 10, where PlanetScope imagery complements Sentinel-2.

(1) First, double acquisitions of Sentinel-2 and PlanetScope during the same day can be used to determine the debris’s short-term surface drift direction. It shows one PlanetScope with a corresponding Sentinel-2 image over Accra, Ghana, on 30^th^ of October 2018, with 4 min and 32 s time difference. Both models detected marine debris, as visible in the probability map.
(2) Second, daily PlanetScope imagery can be used to gap-fill the periods in which the weekly Sentinel-2 imagery is unavailable. This is demonstrated in Figure 10B, where the deployed targets from the Plastic Litter Project 2022 are predicted from Sentinel-2 and PlanetScope imagery with the Unet++ model. The Sentinel-2 images are available only on July 16^th^ and 21^st^. Daily PlanetScope imagery can fill this temporal gap and enable continuous monitoring of the deployed targets at a higher spatial, but lower spectral resolution. We can see that the 4-channel model successfully predicts marine debris for the rectangular 5m × 5m inflatable PVC target deployed during the Plastic Litter Project. The two circular (7m diameter) HDPE-mesh targets are not detected.

Thanks to these two examples, we emphasize that the UNet++ model in our Marine Debris Detector trained on Sentinel-2 imagery worked with PlanetScope images without explicitly having seen annotated PlanetScope imagery. This highlights the broader applicability of the Unet++ model on both satellite modality and the synergy between PlanetScope and Sentinel-2 satellite constellations for marine debris detection.

## Discussion

This work presented and evaluated a training strategy including a dataset, targeted negative sampling, and a segmentation model to automatically identify marine debris of human or natural origins with readily available Sentinel-2 imagery. Our main contribution is the aggregation and harmonization of all annotated Sentinel-2 data for marine debris detection available today. We designed a sampling rule to gather many diverse negative examples and a refinement module to automatically improve hand annotations present in current datasets, which yields a combined training dataset in which deep learning models achieve the best results across different model architectures. The model performances were compared quantitatively and qualitatively on evaluation scenes where the visible marine debris in these scenes is highly likely to contain plastic pollutants. The performance improvements observed are consistent across datasets and model settings. They highlight the importance of designing good datasets for the tasks at hand and prove the necessity to collect, aggregate, and further refine globally distributed datasets of marine debris in future research.

### Role of atmospheric correction

Atmospheric correction with Sen2Cor has proven beneficial in reducing the number of false-positive examples and improving precision. Still, the detector remained sensitive to marine debris with top-of-atmosphere data, which highlights the sensitivity of the model to marine debris. We believe that reliably detecting marine debris from available satellite data is within reach with more annotation and targeted post-processing, such as automatic masking of clouds, land, and shoreline, which we considered beyond the scope of this work. In this work, we trained the detector with Sentinel-2 images of both top-of-atmosphere (L1C-level) and bottom-of-atmosphere (L2A-level with the Sen2Cor algorithm) to ensure that the final model is capable of detecting marine debris from Sentinel-2 imagery at different processing levels. However, further atmospheric correction specific for coastal and aquatic environments, as with the ACOLITE algorithm,^45^ is likely to improve the detection accuracy further.

### Marine debris as a proxy for marine litter

The detection of marine debris remains a proxy objective targeted toward the long-term goal of enabling continuous monitoring of marine litter including plastics and other anthropogenic pollutants from medium-resolution satellite data. Here, automatically establishing the link between detected marine debris and marine pollution is a key question to be addressed in the future. Similar to related work,^40^ we analyzed social media (Durban scene) and *in situ* studies (Accra scene) on a case-by-case basis to deduce that marine plastics are present in marine debris visible in the satellite scenes. Automating this connection remains a challenge that may require integrating *in situ* knowledge (citizen science, or river monitoring) or a targeted acquisition and analysis of high-resolution imagery. Studies^26^^,^^27^ have demonstrated that plastics are present in marine debris by on-site ship-based collection. This establishes that marine debris detection is a suitable, yet rough, proxy for plastic pollution mapping. Ongoing research^29^^,^^31^^,^^53^ in this field demonstrates that distinguishing anthropogenic marine litter from natural types of debris using only features is possible, but remains challenging and is largely unsolved today. Our work concentrated on the prior step of automating the detection of generic marine debris at a large scale largely based on their geometric shape, which can be seen as a first step preceeding the aforementioned litter types characterization.

### Impact of the datasets on model performance and error

In this work, we harmonized several datasets in different settings and made assumptions to maximize the number of annotated data points to train a deep learning model. This required several design choices and modifications of each dataset, e.g., taking a subset of classes to binarize the dataset, or re-processing images to harmonize the atmospheric correction. In particular, the MARIDA and FloatingObjects datasets follow different underlying definitions of marine debris. While MARIDA^38^ was meant to be multi-class and further distinguished different types of water, FloatingObjects^36^ followed a binary classification scheme with a diverse set of negative examples. In sum, the deep segmentation model’s objective is to minimize the error on all training datasets provided. Hence, the harmonization of each dataset to define a consistent learning signal across the training dataset is crucial for an accurate model that provides consistent predictions. This accuracy and consistency is measured by the performance on the test dataset. Here, we took special care to include only data points where plastics and other pollutants are present in the visible aggregations.

### Impact and mitigation of class imbalance

Across the entire satellite archive, only a few scenes show visible aggregations of marine debris. Within these scenes, only a small fraction of pixels contain marine debris (e.g., in the Durban evaluation scene, with 0.05% of marine debris pixels). This needle-in-the-haystack setting requires a curated strategy for training data where the images showing marine debris and non-marine debris are roughly balanced to train and evaluate models effectively. The dataset used in this work balances the data differently.

(1) The FloatingObjects dataset^36^ randomly samples negative non-debris examples across the entire Sentinel-2 scene from random location; in the refined FloatingObjects dataset (used for validation and evaluation), we manually selected a set of meaningful negative points that confused a simpler random forest classifier;
(2) The MARIDA^38^ dataset provides a fixed set of roughly balanced positive (marine debris) and negative (marine water) patches provided by expert annotations for both marine debris and marine water.

A rough balancing is necessary to obtain meaningful metrics where methods and approaches can be compared: in Table 2, we provide quantitative results on the patch-level balanced evaluation dataset, allowing us to compare methods. However, the high accuracy metrics obtained on these balanced datasets may be misleading and lead to a false impression that this problem is solved. To counteract this, we also provided an experiment under a real-world class-imbalanced situation on the Durban study with high imbalance (Section role of atmospheric correction). Here, it became clear that applying the proposed model on an entire Sentinel-2 scene is not perfect and leads to substantial errors and confusion with haze, clouds, ships, and land, as shown in Figure 9, which showed lower performances in an atmospherically corrected setting.

### Relevance for algae and sargassum detection

While the evaluation datasets in our work aimed to measure the detector’s sensitivity to marine litter, we see that the model is also sensitive to detections of floating algae patches and sargassum. This sensitivity is inherently connected to the annotations in the training dataset that were made by visually inspecting the FDI^40^ that is derived from the Floating Algae Index.^41^ Hence, exploration and modification of the training framework presented in this work and initialization from model weights and fine-tuning toward detecting patches of algae and sargassum would be an interesting follow-up work in an active research field.^60^^,^^61^

### Transfer to other satellite products

The synergy of Sentinel-2 with daily available PlanetScope (or other high-resolution imagery) is particularly suitable for further analysis of detected debris and establishing a connection to marine litter. Large-scale monitoring with commercial high-resolution imagery may be infeasible due to the high image acquisition costs. However, selecting a few images with PlanetScope in locations where a Sentinel-2 detector has identified potential marine debris appears feasible. We explored this transferability in Section transferability to PlanetScope resolution where a model trained on 4-channel Sentinel-2 imagery was still sensitive to marine debris in (downsampled) PlanetScope data. Targeted model training on annotated PlanetScope data will likely improve this performance further, which we leave for future work.

### Spatial and spectral features

A further direction to be explored is the heterogeneous composition of objects in marine debris, which varies depending on circumstances (e.g., Flood event in Durban) or the general pollution of the area (Accra scene). This heterogeneity in spectral response further emphasizes the importance and descriptiveness of the shape and geometry in marine debris, which often form elongated lines due to oceanic processes, such as windrows and waterfronts. Further, the geometry of objects is also a suitable descriptor to exclude a variety of negatives, such as ships, clouds, coastline, and wakes, that can have similar spectral responses (e.g., a high FDI index) to marine debris but are distinguishing from marine debris by spatial context. In particular, convolutional neural networks are suitable to learn these patterns in their filter banks if they are trained with large annotated datasets with a diverse set of negative examples.

### Limitations of the study

From the aforementioned discussed topics of class imbalances, the definition of marine debris, and the role of atmospheric correction, it becomes clear that the systematic detection of generic marine debris, individual plastics, or other pollutants remains challenging. Even though we showed that a deep learning model trained with our setup has fewer false-positive predictions than previously proposed models,^36^^,^^38^ the estimated precision on the challenging Durban scene is still low at 22% (136 marine debris among 609 detections). Common confusions are haze, land, and open water, as we evaluated in Section role of atmospheric correction. While false positives over haze and land can be filtered by post-processing, false detections on pure water remain a problem. Another limitation is that we can only detect generic marine debris due to a lack of training data containing confirmed plastic materials. Here, our model can serve as a first filter step to identify suspected plastic areas. We have shown that the trained deep learning model is sensitive to plastic pollutants by evaluating on areas with confirmed plastic outwash, but further work is necessary to separate generic marine debris of natural origins from plastics and other pollutants. Overall, our work lays out a path toward operational detectability of marine litter in the future and further work is necessary to bring this approach to an operational level.

### Conclusion

Remote sensing combined with current machine learning frameworks has the potential to become an efficient and reliable tool to monitor large marine areas.^8^ Still, the data quality used to learn detection models is paramount. We are confident that automated detection of marine debris with satellite remote sensing imagery will provide a repeatable low-cost technology to detect and quantify the level of marine pollution on our planet. Automated detection and quantification will be necessary to inform clean-up operations and measure local policy decisions’ effect. Identifying and quantifying pollution hotspots and addressing the drivers and sources are crucial to creating a cleaner environment for plant, animal, and human life in a sustainable future. Still, further efforts are needed in data collection and on-site validation to build models that can reliably estimate the level of marine pollution from readily available satellite data in a completely automated way. To reach this goal, the lack of reliable annotations and the vagueness and heterogeneity regarding the definition of marine debris at a remote sensing relevant scale remain major limiting factors, which must be improved in future works that include further annotations, potentially informed by crowdsourcing. And even more importantly, in the quantitative comparison provided in this work, it became absolutely clear that the increased model accuracy can be more related to a harmonized dataset rather than to the individual model architectural choices. In this research, we made a step toward automated satellite-based monitoring of marine pollution by detecting marine debris in coastal waters. We hope this work helps accelerate the progress toward large-scale marine litter monitoring within the canon of *trans*-disciplinary machine learning, remote sensing, and marine science research.

## STAR★Methods

### Key resources table

**Table:** 

REAGENT or RESOURCE	SOURCE	IDENTIFIER
**Deposited data**		

FloatingObjects Dataset	Mifdal et al., 2021^36^	https://github.com/ESA-PhiLab/floatingobjects
MARIDA Dataset	Kikaki et al., 2022^38^	https://zenodo.org/records/5151941#.YfFZ_PXP30o
S2Ships	Ciocarlan et al., 2021^46^	https://github.com/alina2204/contrastive_SSL_ship_detection
Refined Floating Objectis	this paper	https://github.com/marccoru/marinedebrisdetector
PlanetScope Scene IDS	this paper	20181031_101439_0f36_3B_AnalyticMS_SR (Figure 10A) 20220717_080454_14_2464_3B_udm2 (Figure 10B) 20220718_083743_48_249days_3B_udm2 (Figure 10B) 20220719_080110_36_2457_3B_udm2 (Figure 10B)

**Software and algorithms**		

Python v3.8.10	Python Software Foundation	https://www.python.org
Pytorch v1.13.1	PyTorch Foundation	https://pytorch.org/
Pytorch Lightning v1.8.2	Lightning AI	https://lightning.ai/
Google Earth Engine	Google	https://earthengine.google.com/

**Other**		

Sentinel-2	Copernicus Program	https://sentinels.copernicus.eu/
PlanetScope	Planet Labs	https://planet.com

### Resource availability

#### Lead contact

Further information and requests for resources should be directed to and will be fulfilled by the lead contact, Marc Ruβwurm (marc.russwurm@wur.nl).

#### Materials availability

The Sentinel-2 image data used in this work is freely available. The PlanetScope data of Figure 10 are not freely available, but can be acquired through an Education and Research Program. The image IDs used in this work are 20181031_101439_0f36_3B_AnalyticMS_SR, 20220717_080454_14_2464_3B_udm2, 20220718_083743_48_249days_3B_udm2, 20220719_080110_36_2457_3B_udm2.

#### Data and code availability

(1) All original code has been deposited at GitHub and is publicly available under https://github.com/MarcCoru/marinedebrisdetector
(2) All Sentinel-2 data used in this work can be downloaded via the data page doc/data.md in the provided GitHub repository. This page lists the MARIDA,^38^ FloatingObjects,^36^ S2Ships^46^ alongside the images of the Plastic Litter Project.^35^^,^^55^ Our annotations in RefinedFloatingObjects are publically available on this page.
(3) Any additional information required to reanalyze the data reported in this paper is available from the lead contact upon request.

### Method details

We train Unet and Unet++ models with a learning rate of 0.01 and weight decay $1\times 10^{-6}$ for 100 epochs in Pytorch using the Pytorch Lightning package. The Unet implementation in this work has 31 million trainable parameters, while the Unet++ has 26 million parameters. Regarding the label refinement module (Section floatingObjects), we compute multiple refined segmentation masks with different parameters and choose a buffer size of 0, 1, or 2 pixels, the β-parameter of the random walker (a penalization coefficient for the walker motion) of 1 or 10, and the marker density for marine debris of 5%, 25%, 50% or 75% (the density of other markers is fixed at 5%). Combined with the original mask, this yields 25 different target masks consistent with the hand annotations and the FDI image but of varying shapes and sizes, as shown in the bottom row of Figure 2. During training, we choose one of these target masks randomly, which, in our opinion, reflects best the undefined borders of the marine debris that we aim to detect and acts as a form of natural label-data augmentation. During training, we monitor the area under the ROC curve (AUROC) on the refined FloatingObjects dataset (Section refinedFloatingObjects) and MARIDA validation set. We store the model weights each time the highest (best) validation AUROC has been reached. We observe that the model systematically underestimates the probability of marine debris due to a heavy class imbalance in the training data. This results in a low precision but high recall when we assign the class marine debris for probability scores above 0.5. We counteract this imbalance by calibrating the classification threshold to balance precision and recall on the validation set. For the Unet++ model, we trained models from different random seeds with validation-optimal thresholds of 0.132 0.0639, and 0.0254 during the experiments shown in this paper. For the Unet, the thresholds were 0.0895, 0.0712, and 0.0643. Training an Unet++ and Unet took eight and 9 h on an NVIDIA RTX 3090 graphics card with multi-threaded data loading with 32 workers. The estimated carbon footprint for one model training run was 2.8 kg.eCO^2^.
